# Marine Waste Management: A Framework for Identifying, Prioritizing, and Resolving Key Challenges

**DOI:** 10.12688/f1000research.168078.1

**Published:** 2025-12-18

**Authors:** Sitikantha Sahoo, Deepak Singhal, Sushanta Tripathy

**Affiliations:** 1 School of Mechanical Engineering, Kalinga Institute of Industrial Technology, Bhubaneswar, Odisha, India

**Keywords:** Shipbuilding, Ship repair, waste, challenges, solutions, Fuzzy Delphi, BWM

## Abstract

**Background:**

The shipbuilding and ship-repair industries are important contributors to world trade. However, it also generates various types of waste, similar to any other industry, which has a detrimental impact on the environment. This study aims to understand the critical need for effective and appropriate waste management practices in this industry by investigating the factors involved in generating various types of waste. It identifies and ranks the major challenges in waste management in this industry. The study also suggests potential solutions for shipyards and repair yards to address these challenges.

**Methods:**

The study used the Fuzzy Delphi Method (FDM) to prioritize the challenges identified through a literature review and finalized them based on the feedback of experts. Further, a list of solutions for these challenges was prepared based on interviews with experts in this area. Finally, the Best Worst Method (BWM) was used to rank the solutions for each of the identified challenges.

**Results:**

The findings indicate that the major challenges for the industry are the generation of hazardous waste and the high costs involved in adopting sustainable practices. The results also showed that strict waste segregation and containment are the top solutions to handle the challenge of hazardous waste generation. Further, the leverage on external funding mechanisms and implementation in a phase-wise manner are the top two solutions to deter the challenge of high costs associated with adopting sustainable practices.

**Conclusions:**

By prioritizing these challenges and solutions, this study provides a road map for shipyards around the world to effectively allocate resources, improve environmental performance, meet regulations, and improve operational performance. This is important for the adoption of an environmentally friendly and economically sustainable shipbuilding and repair industry.

## 1. Introduction

The shipbuilding industry includes the construction and repair of ships. This industry is very important as part of the world’s trade. This industry also faces serious environmental problems due to the generation of large amounts of different types of waste, including sewage, volatile organic compounds, and tributyltin ( Forde et al., 2024). In addition, the lack of a comprehensive law for the regulation of marine waste and inconsistent environmental standards between countries is detrimental to effective marine waste management. Therefore, effective waste management solutions are needed to identify, evaluate, and prioritize waste management problems in shipyards. Shipyards are specialized facilities designed for the construction, repair, and maintenance of all types of vessels. A large variety of activities are carried out in shipyards, including ship design, equipment installation, and various types of tests, all of which require the use of various materials and cause a wide variety of waste ( Samsudin et al., 2022).

Shipyard operations, which are complex and large-scale, result in significant multimedia emissions that affect air, water, and soil quality. In addition, the lack of effective waste control measures in traditional shipbuilding methods causes inefficiencies that result in a decrease in product quality, increase in operating costs, and extension in production time ( Vakili et al., 2023). Therefore, it is essential to find and promote better solutions to waste problems that help protect the environment while improving the economic efficiency of the industry. This research was conducted to find a solution to the environmental problems caused by shipyard waste and to encourage the shipbuilding industry to move in a more sustainable direction. The main objective of this study is to identify and prioritize solutions to the challenges involved in the sustainable management of shipyard waste, along with the identification and ranking of challenges. This will help shipyards know and provide them with tools that are needed to reduce their environmental impact and enhance their operational efficiency. This study starts with a discussion of the various sources of waste generated in shipyards. Moreover, it discusses their harmful effects on the environment.

An emphasis will be placed on a detailed review of current waste management practices and technologies applied in shipyards to assess their efficiency and identify the weak points that should be improved. This will help to set the stage for the development of criteria for the assessment and ranking of sustainable waste management challenges, which will be based on factors such as possible environmental impact, level of economic viability, and level of operational implementation, and will generate a list of challenges that will help shipyards make informed decisions about what challenges to address and which ones to ignore. Furthermore, the prioritization of solutions related to the challenges of sustainable shipyard waste management will help shipyards attain sustainability and improve environmental performance. If shipyards adopt these solutions, they will help preserve natural resources, minimize pollution, improve operational efficiency, and reduce costs for shipyards. Thus, the results of this research are expected to contribute to achieving environmentally responsible and economically viable shipbuilding.

This paper is organized into six main sections. Section 2 reviews the literature, and Section 3 explains the methodology. Section 4 summarizes and discusses the results. Section 5 elaborates on the managerial implications, and Section 6 concludes the paper.

## 2. Literature review

The shipbuilding industry is one of the major industries in global trade. It is also known for one of a major pollutant ( Papamanolis et al. 2018). Different types of waste streams are produced during shipbuilding and repair activities, including asbestos, PCBs, and waste oil. These waste streams are primarily generated during the dismantling of ships ( Du et al. 2018). Ship refitting also causes pollution through the release of nanoparticles, which increases the risks to human health and aquatic life ( López et al., 2022). Therefore, this waste must be managed properly to avoid environmental pollution caused by these operations. The key activities used in shipyard waste management are as follows: (1) appropriate handling of hazardous materials during ship recycling operations ( Du et al., 2018), (2) using lean manufacturing approaches to minimize shipbuilding process waste ( Fitriadi & Ayob, 2022), and (3) using sustainable waste management models and life cycle assessments ( Papamanolis et al., 2018).

Sustainability in shipyards spans waste management to energy and emissions control. DiBarra (2002) introduced the 5S as a cultural change tool towards sustainability. Hossain et al. (2016) addressed environmental degradation from ship breaks in Bangladesh and proposed sustainable management frameworks. Kotrikla (2009) focused on TBT antifouling waste management. Papamanolis et al. (2018) investigated sustainable waste practices in shipyards in Greece. Recent advancements include the work of Vakili et al. (2023), who developed multi-energy and zero-emission frameworks for modern shipyards by using transdisciplinary approaches. Rachmat et al. (2018) and Fitriadi and Ayob (2022) emphasized pollution mitigation and lean practices to reduce shipyard waste. Scipioni et al. (2023) highlighted that the application of circular economy (CE) principles in shipyard activities is essential for achieving sustainability. The CE approach to sustainability includes circular design, end-of-life (EOL) management, sustainability-oriented resource management, eco-design, disassembly design, sustainable procurement, waste reduction and management, and supply chain integration. These strategies are important for achieving social, environmental, and economic value during the shipbuilding process. Thus, CE helps minimize waste and maximize resource efficiency by adopting circular business models and supply chains.

The environmental impact of shipyard activities compelled us to focus on sustainable practices and the green concept of green shipbuilding. The International Maritime Organization (IMO) has been a pioneer in promoting the transition towards zero waste and zero emissions through the development of green ships and green shipyards. The development of environmentally friendly shipyards and the conversion of existing shipyards to meet the stringent requirements of green shipyards ( Koray 2023). The IMO regulations and high environmental consciousness of stakeholders forced the shipbuilding industry to adopt an eco-friendly approach in shipyard operations. The concept of a green shipyard that focuses on minimizing waste and emissions during shipyard activities is evolving in the maritime industry. The green shipbuilding process includes the use of biomaterials and fuels, adoption of renewable energy sources, and naval architecture designs that minimize the environmental impact ( Daud et al. 2024). In addition, the use of sustainable materials, such as composite materials, and energy savings during shipyard activities are also part of green shipbuilding initiatives ( Daud et al., 2024; Dolz et al., 2024). The use of composite materials in shipbuilding is increasing because the use of these materials can lead to material optimization and optimization of the production process optimization ( Dolz et al., 2024). In addition, European Union shipyards are applying new advanced construction methods with both composite and non-composite materials to achieve better production and material optimization. The challenge for the use of these materials is the need for a change in customer perspective to accept life-cycle restrictions, economic limitations, and problems with the recycling of composite materials ( Dolz et al., 2024). Furthermore, effective waste treatment for composite construction must be determined.

In addition, the shipbuilding industry uses industry 4.O technologies to make their processes better and more efficient. These technologies help in controlling and monitoring operations, which in turn leads to a reduction in waste and an overall more efficient operation. The key activities of Industry 4.0 in shipyard operations are as follows: real-time monitoring of operations, automated quality control, optimization of processes using digital twins, and predictive maintenance ( Dolz et al., 2024; Pournara & Konstantinidis, 2021).

## 3. Methodology

This study was conducted using a decision modelling approach to identify and solve shipyard waste management problems. The approach consists of 4 phases as follows: (1) Identification of Challenges in shipbuilding and ship repair waste management, (2) ranking of challenges, (3) formulation of solutions for shipbuilding and ship repair waste management, and (4) Ranking of Solutions.

### 3.1 Identification of challenges

In the first phase of this study, the key challenges of shipyard waste management were identified. First, academic databases such as SCOPUS, Web of Science, and Google Scholar were reviewed. This review focuses on the sources of waste in shipyards, the environmental impact of waste in shipyards, current waste management practices in shipyards, and the problems that hinder effective waste management in shipyards. Keywords such as “shipyard waste,” “shipbuilding waste management,” “ship repair waste,” “marine pollution,” “waste disposal,” “environmentally friendly shipyard practices” were used to search the related literature. In this process, a list of potential challenges is collected. To validate the findings of the literature review and complement the findings from the literature review, we invited three expert panels to provide their feedback. We took their verbal informed consent prior to participation in the study. The purpose of the study, procedures, and participants’ right to withdraw at any time was explained to them during the consent process. Verbal consent was obtained instead of written consent to maintain anonymity of the participants and respect participants’ preference for a more informal process. No expert was under 18 years. The three expert panels were selected based on their experience and knowledge in the field of shipyards, operations, waste management, and environmental science. The selected expert panel consisted of shipyard managers. The results of the expert panel feedback waswere compared with the literature review findings to obtain a validated list of shipyard waste management challenges.

### 3.2 Prioritization of challenges

Recognizing that not all challenges had equal significance, the Fuzzy Delphi Method was employed to determine the relative importance or weight of each identified challenge. The Delphi method is a structured communication technique that relies on a panel of experts to arrive at consensus. The Fuzzy Delphi Method extends this by incorporating fuzzy logic to handle the uncertainty and vagueness inherent in expert judgment. A 7-point Likert scale was used, as outlined in Table 1, for expert input. The average fuzzy numbers (Fna) were calculated using Eq. (1) to determine consensus among experts. The threshold value (d(a,b)) was calculated using Eq. (2) to assess the level of agreement between individual and average fuzzy numbers. Finally, crisp score (Cs) values were determined using Eq. (3) to prioritize the factor according to the process proposed by Jahanvand et al. (2023) and Namayala and Kondo (2024).

$$F_{\mathrm{na}}=b\left(b1,b2,b3\right)=\left[\frac{\sum_{i=1}^{n}a_{1i}}{n},\frac{\sum_{i=1}^{n}a_{2i}}{n},\frac{\sum_{i=1}^{n}a_{3i}}{n}\right]$$ (1)

$$d\left(a,b\right)=\sqrt{\frac{1}{3}\left[{\left(a_{1}-b_{1}\right)}^{2}+{\left(a_{2}-b_{2}\right)}^{2}+{\left(a_{3}-b_{3}\right)}^{2}\right]}$$ (2)

$$C_{s}=\left(\frac{1}{3}\right)*\left(b_{1}+b_{2}+b_{3}\right)$$ (3)

**Table 1.** 7-Point Likert scale and triangular fuzzy score.

Likert scale	Linguistic variable	Triangular fuzzy score
1	Very strongly disagree (VSD)	(0,0,0.1)
2	Strongly disagree (SD)	(0,0.1,0.3)
3	Moderately disagree (MD)	(0.1,0.3,0.5)
4	Agree (A)	(0.3,0.5,0.7)
5	Moderately agree (MA)	(0.5,0.7,0.9)
6	Strongly agree (SA)	(0.7,0.9,1.0)
7	Very strongly agree (VSA)	(0.9,1.0,1.0)

### 3.3 Solution formulation

Based on the challenges identified and ranked in the previous chapter, the third phase of this research was conducted to develop a list of solutions. The main driver to develop the list of solutions in this research was the experts’ interviews. We conducted a series of interviews with the three experts. Each interview focused on a group of challenges. Experts are encouraged to brainstorm and develop a list of solutions based on the best practices and new and innovative technologies in shipyard waste management and management strategies. All solutions proposed by experts were carefully documented. Each solution is described in terms of its description, benefits, implementation requirements, and possible limitations.

### 3.4 Prioritization of solutions

The Best-Worst Method (BWM) was applied to rank the solutions for each challenge. The BWM is a multi-criteria decision-making (MCDM) method, which is a fast and structured method of ranking alternatives. For each challenge, experts should specify the “best” (most preferred) and the “worst” (less preferred) solutions from the set of solutions defined for that challenge. Experts compared the best solution pairwise with all other solutions and all other solutions pairwise with the worst solution on a scale of 1 to 9 (1 indicates that the alternatives are equally preferred, 9 indicates that the difference between the alternatives is as large as possible). A pairwise comparison matrix was used to calculate the weights of the solutions using an optimization solver. The consistency of the pairwise comparison matrix is also examined. Finally, the weights of the solutions for each challenge were used to order the challenges from most preferred to least preferred. Thus, a literature review, expert opinions, and the MCDM method provided solid grounds for the research findings and enabled connecting research findings to shipyard waste management practices.

Step 1: Determination of the Decision-Making Criteria

In this step, a set of decision-making criteria {k1, k2, k3, k4, …, km} necessary to aid the decision towards the objective of the study was determined.

Step 2: Identification of the Best and Worst Criteria

In this step, the assigned decision makers identify the most important criterion (kB) and the least important criterion (kW) from the set of criteria {k1, k2, k3, k4, …, km} without any direct pairwise comparison.

Step 3: Determination of the Preference of the Best Criterion Over All Other Criteria

In this step, decision makers determine the preference of the best criterion (kB) compared to all other criteria (kj, where j = 1,2, …, m). This preference is expressed using a 1–9 point rating scale, as shown in Table 2. The resulting Best-to-Others (BO) vector, denoted as RB, is given by RB = (rB1, rB2, rB3, rB4, …, rBm), where rBj represents the preference for the best criterion (kB) over criterion kj. By definition, when rBB = 1.

**Table 2.** Rating scale for BWM method.

Numerical scale	Linguistic scale
1	Equal importance
2	Somewhat between Equal and Moderate
3	Moderately more important than
4	Somewhat between Moderate and Strong
5	Strongly more important than
6	Somewhat between Strong and Very strong
7	Very strongly important than
8	Somewhat between Very strong and Absolute
9	Absolutely more important than

Step 4: Determination of the Preference of All Other Criteria Over the Worst Criterion

In this step, decision makers determine the preference of all other criteria (ki, where i = 1,2, …, m) compared to the worst criterion (kW). This preference was expressed using a 1–9 point rating scale. The resulting others-to-worst (OW) vector, denoted as RW, is given by RW = (r1W, r2W, r3W, r4W, …, rmW), where riW indicates the preference of criterion ki over the worst criterion (kW). By definition, when rWW = 1.

Step 5: Computation of the Optimal Weights

The optimal weights of the criteria (w1*, w2*, w3*, w4*, …, wm*) should satisfy the conditions; for each pair of wB/wj and wi/wW, the best possible solution is, where wB/wj = rBj and wi/wW = riW. Hence, to obtain the best possible solution, the maximum absolute difference should be minimized among the set of {|wB−rBj*wj|, |wi−riW*wW|}, and the problem can be expressed as follows:

minξ

Subject to:

$$|\mathrm{wB}-{\mathrm{rBj}}^{*}\mathrm{wj}|\;\leq \;\xi \;\text{for}\;\mathrm{all}\;j$$

$$|\mathrm{wi}-{\mathrm{riW}}^{*}\mathrm{wW}|\;\leq \;\xi \;\text{for}\;\mathrm{all}\;i$$

$$\sum_{1}^{m}w_{j}=1$$

wj≥0forallj

The optimal weights (w1, w2, w3, w4, …, wm∗) and consistency indicator ξ∗ can be obtained by solving the above linear programming (LP) problem. The notation ξ∗ indicates the consistency of comparisons. If ξ∗ is closer to zero, it indicates a more consistent and reliable comparison, and vice versa.

## 4. Results and Discussion

The present study utilized fuzzy decision modelling to prioritize the challenges in marine waste management within shipbuilding and ship repair yards. Table 3 presents the evaluation of expert feedback, including the threshold values (d), percentage of expert agreement (Pe), crisp scores (Cs), and the decision to accept or reject the identified influencing factors. The factors were ranked in descending order based on their crisp scores. The threshold values (d) for all factors were below 0.2, indicating strong expert consensus. Additionally, all factors exceeded the 75% expert agreement threshold and had defuzzification values (Cs) above 0.5, according to the criteria outlined by Al-Rikabi and Montazer (2024). These results confirm expert consensus on the identified challenges in shipyard waste management. Finally, the influencing factors were prioritized based on their rankings, as shown in Table 3 and presented in Table 4.

**Table 3.** Results from the evaluations of Experts’ Opinions.

Sl No.	Influencing factors	Threshold value ( *d*)	% of expert agreement ( *P _e_*)	Crisp score ( *C _s_*)	Decision (Accept/Reject)	Rank
1	Wastewater Management	0.195	91	0.91	Accept	3
2	Compliance with Environmental Regulations	0.121	91	0.707	Accept	6
3	Waste from Decommissioning/refitment/mid-life upgrade etc	0.161	100	0.904	Accept	4
4	Non-Degradable Materials	0.107	81.9	0.879	Accept	5
5	Mixed Waste Streams	0.166	81.9	0.537	Accept	9
6	Hazardous Waste Generation	0.031	100	0.964	Accept	1
7	Lack of Skilled Workforce	0.133	91	0.664	Accept	8
8	Limited Space for Waste Storage	0.082	100	0.682	Accept	7
9	Large-Scale Scrap Metal Management	0.126	81.9	0.519	Accept	10
10	High Cost of Sustainable Practices	0.132	91	0.934	Accept	2

**Table 4.** Prioritized challenges.

Influencing factors	Rank
Wastewater Management	3
Compliance with Environmental Regulations	6
Waste from Decommissioning/refitment/mid-life upgrade	4
Non-Degradable Materials	5
Mixed Waste Streams	9
Hazardous Waste Generation	1
Lack of Skilled Workforce	8
Limited Space for Waste Storage	7
Large-Scale Scrap Metal Management	10
High Cost of Sustainable Practices	2

The results show that hazardous waste generation is the top challenge in shipbuilding and ship repair yards, followed by the high cost of sustainable practices, wastewater management, waste from decommissioning/re-fitment/upgrades, non-degradable materials, compliance with environmental regulations, limited space for waste storage, lack of skilled workforce, mixed waste streams, and large-scale scrap metal management. Addressing these issues is crucial for achieving sustainable industrial operations.

Tables 5- 14 shows the list of solutions identified with the input of experts and the ranking of the solutions obtained by applying the steps of the BWM method mentioned in the section. Table 5 shows the list of solutions and ranking of the solutions related to the challenge of “hazardous waste generation”. The results show that waste segregation and containment (A1) are the best solutions to this challenge. Therefore, better techniques for waste segregation and containment are required to avoid environmental contamination. Staff training and contingency (A2) were in the second position in the ranking list. This solution leads to safe handling and an emergency response. Monitoring and compliance (A3) and eco-friendly materials (A4) are in the third and fourth positions, respectively. Monitoring and compliance through tracking and audits control hazardous waste generation. Additionally, eco-friendly materials must be substituted to reduce waste generation. Energy recovery and disposal (A5) is at the last position in the ranking list. The mechanisms of sustainable energy recovery and disposal are environmentally friendly solutions for waste that cannot be avoided. All of these solutions are coherently connected to form a robust framework for hazardous waste management.

**Table 5.** Solutions for Hazardous waste generation.

Challenge	Solutions	Weight obtained from BWM method	Ranking
Hazardous Waste Generation	Waste Segregation & Containment (A1)	0.362	1
	Staff Training & Contingency (A2)	0.289	2
	Monitoring & Compliance (A3)	0.193	3
	Eco-Friendly Materials (A4)	0.082	4
	Energy Recovery & Disposal (A5)	0.072	5

**Table 6.** Solutions for large-scale scrap metal management.

Challenge	Solutions	Weight obtained from BWM method	Ranking
Large-Scale Scrap Metal Management.	Sorting & Segregation (B1)	0.367	1
	Advanced Recycling (B2)	0.294	2
	Digital Inventory Control (B3)	0.147	3
	Circular Economy Integration (B4)	0.117	4
	Occupational Safety (B5)	0.073	5

**Table 7.** Solutions for non-degradable materials.

Challenge	Solutions	Weight obtained from BWM method	Ranking
Non-Degradable Materials	Material Substitution (C1)	0.500	1
	Collection & Segregation (C2)	0.222	2
	Recycling & Valorization (C3)	0.167	3
	Educational & Digital Oversight (C4)	0.112	4

**Table 8.** Solutions for constrained capacity for waste storage.

Challenge	Solutions	Weight obtained from BWM method	Ranking
Constrained Capacity for Waste Storage	Compaction & Space Optimisation (D1)	0.525	1
	Environmental Protection & Design (D2)	0.187	2
	Just-in-Time Waste Removal (D3)	0.186	3
	Outsourcing & Partnerships (D4)	0.101	4

**Table 9.** Solutions for compliance with environmental regulations.

Challenge	Solutions	Weight obtained from BWM method	Ranking
Compliance with Environmental Regulations	Compliance Framework (E1)	0.370	1
	Monitoring & Reporting (E2)	0.288	2
	Proactive Measures (E3)	0.144	3
	Staff Awareness (E4)	0.115	4
	Continuous Improvement (E5)	0.082	5

**Table 10.** Soutions for wastewater management.

Challenge	Solutions	Weight obtained from BWM method	Ranking
Wastewater Management	Wastewater Treatment Systems (F1)	0.4	1
	Closed-Loop Water Recycling (F2)	0.227	2
	Scalable Treatment Solutions (F3)	0.152	3
	Employee Training & Digital Tools (F4)	0.151	4
	Environmental & Regulatory Benefits (F5)	0.068	5

**Table 11.** Solutions for higher costs of sustainable practices.

Challenge	Solutions	Weight obtained from BWM method	Ranking
Higher Costs of Sustainable Practices	Leveraging Government Incentives (G1)	0.292	1
	Phased Implementation (G2)	0.208	2
	Circular Economy Models (G3)	0.139	3
	Public-Private Partnerships (G4)	0.104	4
	Workforce Training (G5)	0.083	5
	Strategic Outsourcing (G6)	0.069	6
	Long-Term Cost-Benefit Analysis (G7)	0.059	7
	Financial Burdens (G8)	0.041	8

**Table 12.** Solution for insufficient skilled labor force.

Challenge	Solutions	Weight obtained from BWM method	Ranking
Insufficient Skilled Labor Force	Establishing Comprehensive Training Programs (H1)	0.287	1
	Apprenticeship & Vocational Training Partnerships (H2)	0.217	2
	Defining Clear SOPs (H3)	0.144	3
	Improve Workforce Retention Practices (H4)	0.109	4
	Minimizing Human Labor Dependence by Automation (H5)	0.108	5
	Government & Industry Workforce Development Support (H6)	0.086	6
	Long-Term Workforce Planning & Knowledge Transfer (H7)	0.046	7

**Table 13.** Solution for heterogeneous waste streams.

Challenge	Solutions	Weight obtained from BWM method	Ranking
Heterogeneous Waste Streams	Establishing Source Segregation Systems (I1)	0.314	1
	Workforce Training and Incentive Programs (I2)	0.220	2
	Developing Standardized Procedures for Waste Disposal (I3)	0.146	3
	Waste Minimization Strategies (I4)	0.110	4
	Monitoring and Continuous Improvement (I5)	0.088	5
	Combining Automated Sorting Methods (I6)	0.073	6
	Just-In-Time Waste Collection and Treatment (I7)	0.047	7

**Table 14.** Solution for waste from decommissioning operations.

Challenge	Solutions	Weight obtained from BWM method	Ranking
Waste from Decommissioning Operations	Hazardous Waste Identification and Containment (J1)	0.317	1
	Efficient Waste Classification and Organization (J2)	0.203	2
	Advanced Techniques of Recycling and Waste Recovery (J3)	0.135	3
	Logistical Optimisation and Regulation Compliance (J4)	0.102	4
	Developing Specialized Decommissioning Sites (J5)	0.101	5
	Sustainable Decommissioning Practices (J6)	0.081	6
	Continuous Improvement and Tracking (J7)	0.057	7

Table 6 shows the list of solutions and ranking of solutions related to the challenge “Large-Scale Scrap Metal Management.” The results show that sorting and segregation (B1) are the most important solutions for tackling this challenge. It is essential to implement efficient sorting and material segregation procedures to maximize the value and purity of recovered materials. Advanced recycling (B2) occupies the second place in the ranking list. The adoption of advanced recycling and processing technologies enhances resource recovery and minimizes waste. Digital inventory control (B3) was in third place in the ranking list. Digital inventory control and economic optimization tools provide real-time data and facilitate informed decision-making. Circular economy integration (B4) is in fourth place. The integration of circular economies sustainably promotes resource efficiency and reduces the reliance on virgin materials. Thus, it provides a more environmentally responsible and economically viable system. Occupational safety (B5) ranked last in the ranking list. Occupational safety considerations ensure a secure work environment for all personnel involved in the process.

Table 7 shows the list of solutions and ranking of solutions related to the challenge “Non-degradable materials.” The material substitution (C1) occupies the first position. The use of recyclable and biodegradable materials through supplier collaboration is key to addressing this challenge. Collection and segregation (C2) was in second place. The implementation of effective organized collection and segregation mechanisms boosts recycling. Recycling and valorization (C3) are in the third position in the ranking list. The use of effective recycling and waste vaporization technologies minimizes landfill use. Finally, educational initiatives and digital oversight (C4) are in the fourth position. This is vital for proper handling and process improvements. This integrated approach, which focuses on education, efficient handling, advanced technology, and material choices, is necessary to reduce the environmental burden of nondegradable materials.

Table 8 shows the list of solutions and ranking of solutions related to the challenge “Constrained Capacity for Waste Storage.” Compaction and space optimization (D1) occupy the top positions in the ranking list. Strategic compaction and space optimization strategies, such as using compactors and stackable units, maximize the existing space. Environmental protection and design (D2) are in the second position. Environmental protection measures and infrastructure design, such as impermeable floors and vertical storage, ensure safety and efficient space utilization. Just in time, waste removal (D3) lies at the third position. The implementation of just-in-time waste removal and predictive digital tracking ensures collection with production and prevents overflow. Outsourcing and partnership (D4) were the last places in the ranking list. However, outsourcing strategies and regulatory compliance partnerships offer on-site treatment. It minimizes on-site space requirements while ensuring adherence to regulations.

Table 9 shows the list of solutions and ranking of solutions related to the challenge “Compliance with Environmental Regulations.” The compliance framework (E1) rests at the top of the list. Environmental regulatory compliance in shipyards demands a proactive and organized strategy that encompasses regulations related to waste, emissions, and records. Moreover, it involves the development of a compliance framework aligned with international and national conventions and laws. Monitoring and reporting (E2) ranked second on the list. Robust monitoring and reporting systems, such as electronic tools and audits, are crucial for addressing this challenge. Proactive measures (E3) are in third position. Proactive measures to reduce environmental impacts, such as eco-friendly materials and clean energy, should be embraced. Staff awareness (E4) was in the fourth position. The cultivation of staff awareness and training in regulations and procedures is vital for mitigating this challenge. Finally, the last position was a continuous improvement. It is essential to avoid these repercussions and foster sustainability.

Table 10 shows the list of solutions and ranking of solutions related to the challenge of wastewater management. Effective wastewater management in shipyards is vital to environmental protection and operational efficiency. The results show that waste water treatment (F1) occupies the top position in the ranking list. The implementation of advanced wastewater treatment systems, such as oil-water separators and chemical plants, is essential for purifying pollutants before discharge. Closed-loop water recycling (F2) is placed in the second position. Therefore, it is essential to reduce the wastewater volume. The scalable treatment solutions (F3) are in the third position. Scalable treatment solutions such as modular units and external contracts are required to address seasonal variations. Employee training and digital tools (F4) were placed in fourth position. This enhances proper handling and ensures adherence to regulations. Environmental and regulatory benefits (F5) were in the last position. These integrated solutions yield significant environmental and regulatory benefits, which promote long-term sustainability.

Table 11 shows the list of solutions and ranking of solutions for the challenge “Higher Costs of Sustainable Practices.” Eight solutions were identified and prioritized using the proposed methodology. Leveraging government incentives (G1) occupied the top position in the ranking list. Government support is essential for offsetting initial investments. Phased implementation (G2) is in second position. Modular investments allow for gradual adoption. Circular economy models (G3) are in third position. The use of cost-saving circular economy models through reuse and recycling minimizes the long-term expenses. Public-private and industry partnerships (G4) are in the fourth position. This facilitates resource- and knowledge-sharing. Workforce training (G5) was in the fifth position. Investment in workforce training and process improvement enhances efficiency. Strategic outsourcing (G6) is in sixth position. Strategic outsourcing can reduce capital expenditures. The Long term cost-benefit analysis is in the seventh position. A long-term cost-benefit analysis revealed the economic and regulatory advantages of sustainable practices. Finally, financial burden (G8) occupies the last position in the ranking list. The incorporation of all eight proposed solutions will help mitigate this challenge.

Table 12 shows the list of solutions and ranking of these solutions for the challenge “Insufficiently Skilled Labor Force”. Seven solutions were proposed with the help of experts, and ranked using the proposed methodology. The establishment of comprehensive training programs (H1) was the first position. It includes conferences, seminars, on-the-job training, workshops, and e-learning. Apprenticeship and vocational training partnerships (H2) are in the second position, and shipyards must leverage them to ensure a continuous talent pipeline. Clear standard operating procedures (H3) occupy the third position, and the implementation of visual aids guides employees. Improved workforce retention practices (H4) are placed in the fourth position, which is vital for competitive benefits and growth opportunities. Minimizing human labor dependence (H5) occupies the fifth position, which is necessary because automation enhances efficiency. Government and industry support (H6) is placed in the sixth position, and support for workforce development can offset training costs. Long-term workforce planning and knowledge transfer (H7) occupy the seventh position, and their implementation ensures sustained expertise.

Table 13 shows the list of solutions and ranking of solutions related to the challenge “Heterogeneous Waste Streams.” For effective shipyard waste management, it is essential to manage heterogeneous waste streams. Seven solutions are identified and ranked using the proposed methodology. Establishing Source Segregation Systems (I1) has emerged as the top solution to heterogeneous waste stream challenges. Workforce training and incentive programs (I2) were in the second position. This ensured employee participation. Standardized procedures for waste disposal (I3) are in third position. The shipyards must ensure the formation and implementation of these standard procedures. Waste minimization strategies (I4) occupy the fourth position, and it is necessary to reduce their complexity. Monitoring and continuous improvement (I5) lies in the fifth position and optimizes the entire process through audits and feedback. Combined automated sorting methods (I6) occupy the sixth position, and it is necessary to enhance their efficiency. Just-in-time waste collection and treatment (I7) is placed in the last position, which helps prevent contamination. Partnerships with specialists in waste management organizations provide expertise in complex streams.

Table 14 shows the list of solutions and the ranking of solutions for the challenge “waste from decommissioning operations.” Decommissioning waste management requires an integrated approach. Hazardous waste identification and containment (J1) has emerged as the top solution, which implies thorough assessment and secure storage of waste. Efficient waste classification and organization (J2) at the second position emphasizes on-site segregation and decontamination, which further improves recycling. Advanced techniques of recycling and waste recovery (J3) occupy a third position and help in resource reclamation. Logistical optimization and regulation compliance (J4) via real-time monitoring and training are essential for solving the problem of decommissioning waste. The development of specialized decommissioning sites (J5) occupies the fifth position, which implies that appropriate infrastructure enhances efficiency and compliance. Sustainable decommissioning practices (J6) are placed at the sixth position in ranking, and embracing them minimizes environmental harm. Continuous improvement and tracking (J7) occupies the last position and optimizes the entire process through audits and advanced technology.

## 5. Implications

The findings of this research provide significant implications for shipbuilding and ship repair industries by prioritizing waste management challenges and ranking potential solutions. First, the findings showed that the identification of hazardous waste generation and the high cost of sustainable practices are the top two challenges. This underscores the immediate need for solutions that address both the environmental risks and economic feasibility associated with these two challenges. The highest rank of hazardous waste generation implies the need for robust identification, containment, and safe disposal strategies, as shown in Table 5. Similarly, Table 11 shows that a focus on cost-saving opportunities and leverage in external funding mechanisms is required to deter the challenge of high costs associated with adopting sustainable practices.

Second, wastewater management and waste from decommissioning and re-fitment rank in 3rd and 4th position respectively. This shows that the environmental impact and waste in these two areas are relatively significant. The solutions for these two types of waste problems ranked in 3rd and 4th position are presented and ranked in Tables 10 and 14, respectively, which can provide more guidance for shipyards to deal with these two types of waste. Third, middle-ranking challenges from the list, such as Non-Degradable Materials and Environmental Regulations Framework (ranked in 5th and 6th position), still need to substitute materials more actively, develop more recycling technologies, and build more environmental regulation frameworks, which are presented in Tables 7 and 9. The solutions presented here can direct shipyards to address these two problems. Finally, the waste management challenges ranked in the lower part, such as Limited Space for Waste Storage, Skilled Workforce Deficiency, Mixed Waste Streams, and Large-Scale Scrap Metal Waste Management, may not be as serious as the above problems, according to the opinions of the experts. However, the solutions presented in Tables 4, 6, 10 and 11 still provide specific strategies for shipyards to make full use of space, cultivate human resources, sort waste, and deal with scrap metals.

The ranking of solutions within the same challenge offers shipyards a ranked list of things to do. For example, waste segregation and containment are ranked first as the most useful solutions for hazardous waste generation. This implies that shipyards should focus on implementing or improving systems to separate and contain hazardous materials safely. Leveraging government incentives is ranked first for the challenge of the high costs of sustainable practices. This implies that shipyards should focus on finding external funding to support the initial costs. In summary, this research provides guidelines for shipyards to strategically allocate their resources and implement solutions to waste management issues. By focusing on the challenges ranked at the top of the list and implementing the solutions ranked at the top of the list, the shipbuilding and ship repair industry can begin making substantial advances towards environmental and regulatory sustainability, as well as operational efficiency. The results also show that these challenges are interconnected, and that integrated waste management is necessary.

## 6. Conclusion

This study thoroughly examined the complex challenges associated with managing marine waste in shipbuilding and repair industries around the world. The analysis utilized robust decision modelling techniques to first identify the most challenging problems associated with marine waste using the Fuzzy Delphi Method, and then rank the proposed solutions using the Best-Worst Method. This study contributes significantly to the knowledge base of a critical trans-industry challenge that affects shipbuilding and repair companies around the world. The ranking of hazardous waste generation and the high cost of sustainable waste management as the two most challenging problems provides a clear focus for immediate attention to address the dual challenges of environmental and economic impacts on operations for shipbuilding companies around the world.

The results of this study clearly highlight the fundamental need for strict compliance with waste segregation and containment practices for the effective and efficient management of hazardous waste and other materials used in shipyards worldwide. The economic realities of operations for all shipyards in making their operations more sustainable require a strategic focus on leveraging available financial incentives, a phased implementation approach, and collaborative approaches to share costs and facilitate technology transfer and knowledge sharing for success for all shipyards around the world.

The large volumes and environmental impacts of wastewater and other operations related to decommissioning/re-fitment/mid-life upgrades for all shipyards worldwide requires a focus on the use of new treatment technologies and strict protocols for handling the specific types of pollutants that are common in each region worldwide. The results of this study provide a clear focus on the problems and ranked set of solutions for all shipyards around the world to adopt for their operations. By addressing the most challenging problems and implementing the highest-ranked solutions for success, the international community of shipbuilders and repairers can make significant progress in addressing the need for environmentally friendly operations and practices. The results of this study will be a valuable resource for all around the world to reduce their environmental footprint and improve their overall operational effectiveness for their companies.

## Ethics statement

Ethical approval was not required for this study as it did not involve clinical interventions, vulnerable populations, or sensitive personal data.

## Data Availability

1. Figshare. Raw Data for FDM. doi.org/10.6084/m9.figshare.30653672 ( Sahoo, Sitikantha et al., 2025a). The project contains the following underlying data:
• Response to Fuzzy Delphi.docx. (Raw data for Fuzzy Delphi method also called Decision matrix) Data are available under the terms of the Creative Commons Attribution 4.0 International license (CC-BY 4.0).
2. Figshare. Raw Data for BWM. doi.org/10.6084/m9.figshare.30653969 ( Sahoo, Sitikantha et al., 2025b). The project contains the following underlying data:
• Response to BWM_Questionnaire_R.docx (Raw data for BWM calculations) Data are available under the terms of the Creative Commons Attribution 4.0 International license (CC-BY 4.0). 1. Figshare. Fuzzy Delphi Questionnaire. doi.org/10.6084/m9.figshare.30653618 ( Singhal, Deepak et al., 2025). The project contains the following underlying data:
•
Fuzzy_Delphi_Questionnaire.docx (Questionnaire used for Fuzzy Delphi method) Data are available under the terms of the Creative Commons Attribution 4.0 International license (CC-BY 4.0).
2. Figshare. BWM Questionnaire. doi.org/10.6084/m9.figshare.30653633 ( Sahoo, Sitikantha et al., 2025c). The project contains the following underlying data:
•
BWM_Questionnaire_R.docx (Questionnaire used for Best Worst Method) Data are available under the terms of the Creative Commons Attribution 4.0 International license (CC-BY 4.0).

## References

[ref1] Al-Rikabi YK Montazer GA : Designing an e-learning readiness assessment model for Iraqi universities employing Fuzzy Delphi Method. *Educ. Inf. Technol.* 2024;29(2):2217–2257. 10.1007/s10639-023-11889-0 37361783 PMC10225780

[ref2] Daud AHM Harun M Jeevan J : A systematic review of the current trends and potential practices of green shipbuilding. *Australian Journal of Maritime & Ocean Affairs.* 2024;16(1):105–126. 10.1080/18366503.2023.2194120

[ref3] DiBarra C : 5S—A Tool for Culture Change in Shipyards. *Journal of Ship Production.* 2002;18(03):143–151. 10.5957/jsp.2002.18.3.143

[ref4] Dolz M Martinez X Sá D : Composite materials, technologies and manufacturing: Current scenario of European Union shipyards. *Ships Offshore Struct.* 2024;19(8):1157–1172. 10.1080/17445302.2023.2229160

[ref5] Du Z Zhang S Zhou Q : Hazardous materials analysis and disposal procedures during ship recycling. *Resour. Conserv. Recycl.* 2018;131:158–171. 10.1016/j.resconrec.2018.01.006

[ref6] Fitriadi F Ayob AFM : Identifying the Shipyard Waste: An Application of the Lean Manufacturing Approach. *International Journal of Global Optimization and Its Application.* 2022;1(2):100–110. 10.56225/ijgoia.v1i2.19

[ref7] Forde R Brandão AT Bowman D : Marine waste derived carbon materials for use as sulfur hosts for Lithium-Sulfur batteries. *Bioresour. Technol.* 2024;406:131065. 10.1016/j.biortech.2024.131065 38969241

[ref8] Hossain MS Fakhruddin ANM Chowdhury MAZ : Impact of ship-breaking activities on the coastal environment of Bangladesh and a management system for its sustainability. *Environ. Sci. Pol.* 2016;60:84–94. 10.1016/j.envsci.2016.03.005

[ref9] Jahanvand B Mortazavi SB Mahabadi HA : Determining essential criteria for selection of risk assessment techniques in occupational health and safety: A hybrid framework of fuzzy Delphi method. *Saf. Sci.* 2023;167:106253. 10.1016/j.ssci.2023.106253

[ref10] Koray M : Prioritizing Shipyard Conversion Requirements Regarding Green Ship and Green Shipyard Concept. *Transactions on Maritime Science.* 2023;12(2):658–667. 10.7225/toms.v12.n02.w02

[ref11] Kotrikla A : Environmental management aspects for TBT antifouling wastes from the shipyards. *J. Environ. Manag.* 2009;90:S77–S85. 10.1016/j.jenvman.2008.07.017 18951695

[ref13] López M López-Lilao A Ribalta C : Particle release from refit operations in shipyards: Exposure, toxicity and environmental implications. *Sci. Total Environ.* 2022;804:150216. 10.1016/j.scitotenv.2021.150216 34520930

[ref14] Namayala PP Kondo TS : Application of fuzzy Delphi technique to identify analytical lenses for determining the preparation of free and open source software projects for user experience maturity. *Sci. Comput. Program.* 2024;237:103136. 10.1016/j.scico.2024.103136

[ref15] Papamanolis G Giannakopoulos E Kalavrouziotis IK : Shipyards waste and sustainable management in Greece: case study. *Desalin. Water Treat.* 2018;127:90–96. 10.5004/dwt.2018.22595

[ref16] Pournara A Konstantinidis FK : Shipyards, Shipbreaking industry and the contribution of the Industry 4.0. *17th International Conference on Environmental Science and Technology.* 2021.

[ref17] Rachmat A Emil B Herdis H : Minimize the impact of waste pollution in ship repair processes to Improving Shipyard Industrial Infrastructure Sustainability. *E3S Web of Conferences.* EDP Sciences;2018; Vol.73:08006.

[ref18] Samsudin MS Zainol I Salleh Z : Design of Green Ship Recycling Yards: A Review. *Advanced Materials and Engineering Technologies.* 2022;183–192. 10.1007/978-3-030-92964-0_18

[ref19] Scipioni S Dini G Niccolini F : Exploring circular shipbuilding: A systematic review on circular economy business models and supporting technologies. *J. Clean. Prod.* 2023;422:138470. 10.1016/j.jclepro.2023.138470

[ref20] Vakili S Schönborn A Ölçer AI : The road to zero emission shipbuilding industry: A systematic and transdisciplinary approach to modern multi-energy shipyards. *Energy Conversion and Management: X.* 2023;18:100365. 10.1016/j.ecmx.2023.100365

[ref21] Sahoo S Singhal D Tripathy S : Raw data for FDM.Dataset. *figshare.* 2025a. 10.6084/m9.figshare.30653672.v1

[ref22] Sahoo S Singhal D Tripathy S : Raw data for BWM.Dataset. *figshare.* 2025b. 10.6084/m9.figshare.30653969.v1

[ref23] Singhal D Tripathy S Sahoo S : Fuzzy Delphi Questionnaire.Dataset. *figshare.* 2025. 10.6084/m9.figshare.30156067.v1

[ref24] Sahoo S Singhal D Tripathy S : BWM Questionnaire.Dataset. *figshare.* 2025c. 10.6084/m9.figshare.30156268.v1

