# Supplementary material for: Marine Waste Management: A Framework for Identifying, Prioritizing, and Resolving Key Challenges
Source: F1000Res. 2025 Dec 18;14:1408. [Version 1] doi: 10.12688/f1000research.168078.1 (PMC12887368; doi:10.12688/f1000research.168078.1)
Supplement: Peer review history

# Reviewer report: Reviewer response for version 1

**DOI:** 10.5256/f1000research.185236.r452876

**Published:** 2026-02-09

**Authors:** Ramakrushna Padhy

**Affiliations:** 1 Indian Institute of Management Sambalpur, Sambalpur, Odisha, India

The manuscript presents a well-structured and methodologically sound study that addresses an important and practically relevant problem in shipyard waste management. The authors adopt a **decision modelling approach** that is logical, systematic, and appropriate for the complexity of shipbuilding and ship repair operations.

 The study is clearly organized into **four coherent phases**:

 (1) identification of waste management challenges,

 (2) ranking of these challenges,

 (3) formulation of feasible solutions, and

 (4) ranking of the proposed solutions.

 This phased approach enhances the clarity of the research design and allows readers to easily follow the progression from problem identification to solution prioritization. The integration of ranking mechanisms in both challenges and solutions strengthens the decision-support value of the study and improves its managerial relevance.

 The **language and presentation are clear, concise, and appropriate for an international journal audience**. The arguments are logically developed, and the methodology is adequately explained, enabling replication and practical application. The findings provide actionable insights for practitioners and policymakers involved in shipyard operations and environmental management.

 Overall, the manuscript makes a **meaningful contribution to the literature on sustainable shipyard operations and waste management decision-making**. No major revisions are required.

**Recommendation:** *Accept for indexing*

Is the work clearly and accurately presented and does it cite the current literature?

Yes

If applicable, is the statistical analysis and its interpretation appropriate?

Yes

Are all the source data underlying the results available to ensure full reproducibility?

Yes

Is the study design appropriate and is the work technically sound?

Yes

Are the conclusions drawn adequately supported by the results?

Yes

Are sufficient details of methods and analysis provided to allow replication by others?

Yes

Reviewer Expertise:

Productivity, Sustainability

I confirm that I have read this submission and believe that I have an appropriate level of expertise to confirm that it is of an acceptable scientific standard.

# Reviewer report: Reviewer response for version 1

**DOI:** 10.5256/f1000research.185236.r445452

**Published:** 2026-01-10

**Authors:** Ebru Caymaz

**Affiliations:** 1 Canakkale Onsekiz Mart University, Canakkale, Turkey

First of all, the author(s) elaborately presents the problem within the text. However, background section of the study is recommended to revised.

 It is stated that "This study aims to understand the critical need for effective and appropriate waste management practices in this industry by investigating the factors involved in generating various types of waste. It identifies and ranks the major challenges in waste management in this industry"

 In the main text it is also stated that "The main objective of this study is to identify and prioritize solutions to the challenges involved in the sustainable management of shipyard waste, along with the identification and ranking of challenges"

 The terms of aim and objective are interchangebly used, therefore it should be updated. 

 In terms of methodology, preferred method is quite okay and yet, limitations of the study should also be explained further.

Is the work clearly and accurately presented and does it cite the current literature?

Yes

If applicable, is the statistical analysis and its interpretation appropriate?

I cannot comment. A qualified statistician is required.

Are all the source data underlying the results available to ensure full reproducibility?

Yes

Is the study design appropriate and is the work technically sound?

Yes

Are the conclusions drawn adequately supported by the results?

Yes

Are sufficient details of methods and analysis provided to allow replication by others?

Yes

Reviewer Expertise:

Sustainable development

I confirm that I have read this submission and believe that I have an appropriate level of expertise to confirm that it is of an acceptable scientific standard.
